# An Immature Type II Dens Invaginatus in a Mandibular Lateral Incisor with Talon's Cusp: A Clinical Dilemma to Confront

**DOI:** 10.1155/2014/826294

**Published:** 2014-02-09

**Authors:** Anshul Gangwar, Deepa Singal, K. Y. Giri, Anshita Agarwal, S. Sruthi Keerthi

**Affiliations:** ^1^Department of Pediatric & Preventive Dentistry, Institute of Dental Sciences, Uttar Pradesh Bareilly-243006, India; ^2^Department of Oral & Maxillofacial Surgery, Institute of Dental Sciences, Uttar Pradesh Bareilly-243006, India; ^3^Department of Oral Pathology, Vananchal Dental College & Hospital, Garhwa, Jharkhand-822114, India

## Abstract

Dens invaginatus (DI) is a malformation of teeth probably resulting from an infolding of the dental papilla during tooth development. DI is classified as type I, II, and III by Oehlers depending on the severity of malformation. The maxillary lateral incisor is the most commonly affected tooth. Structural defects do exist in the depth of the invagination pits, and as a consequence, the early development of caries and the subsequent necrosis of the dental pulp, as well as abscess and cyst formation are clinical implications associated with DI. Occasionally, we can see more than one developmental anomaly occurring in a single tooth. In such cases it becomes important to identify the anomalies and initiate a proper treatment plan for good prognosis. In this paper, an unusual case of DI which clinically presented as a huge talons cusp affecting a mandibular lateral incisor tooth is described. This case report illustrates grinding of the talons cusp followed by nonsurgical endodontic management of dens invaginatus type II with an immature apex and periapical lesions, in which Mineral Trioxide Aggregate (MTA) shows a complete periapical healing with bone formation at the site of the lesions.

## 1. Introduction

Dens invaginatus (DI), commonly known as dens in dente, is a developmental anomaly resulting from invagination in the surface of a tooth crown before calcification has occurred. Coronal invaginations usually originate from an anomalous infolding of the enamel organ into the dental papilla. The most extreme form of this anomaly is referred to as “dilated odontome.” This kind of malformation was first described by “Ploquet” in 1794 in whale's tooth [1]. DI was first described as “a tooth within a tooth” by Salter in 1855 [2]. Hallet introduced the term dens invaginatus in order to clarify the point that enamel is located centrally and the dentine peripherally due to the invagination. Since then it has been a preferred term, though dens in dente is a more commonly used term [3]. DI in human tooth was first described by a dentist named Socrates in 1856 [1].

The frequency of its occurrence is 0.04 to 10% of all dental malformations [4]. The permanent dentition is involved three times more commonly than the deciduous dentition. The teeth most affected are the maxillary lateral incisors with a prevalence of 0.25–5.1%, frequently bilateral (43%), followed by central, canines, premolars, and molars. Langlais et al. noted that the mandibular occurrence of this anomaly is rare. The literature review showed only 10 cases involving 13 mandibular teeth with a majority in mandibular incisors among three cases involving four teeth [5].

Synonyms for this malformation are dens in dente, invaginated odontome, dilated gestant odontome, dilated composite odontome, deep foramen caecum, tooth inclusion, dentoid in dente, gestant odontome, and dents telescopes—invagination is the common denominator of these lesions [6, 7].

Another such anomaly is talons cusp. Talons cusp is considered a type of dens evaginatus. W.H. Mitchell was the first to describe it. Mellor and Rippa named it as an accessory cusp. The origin of the name is said to be so because this accessory cusp resembles the talon (tail) of an eagle when seen from an occusal aspect [8].

There are case reports of co-occurrence of multidevelopmental anomalies in a single tooth. A few might give rise to clinical symptoms in early life, with a few manifesting later. The identification and treating the co-existing anomaly is important in the treatment plan for a better prognosis. Here we are reporting a case of Oehlers type II DI which clinically presented as a talons cusp, whose occurrence in the mandibular arch is a rare entity. Prophylactic grinding with root canal therapy using MTA apexification was done.

## 2. Case Report

**Figure 1:**
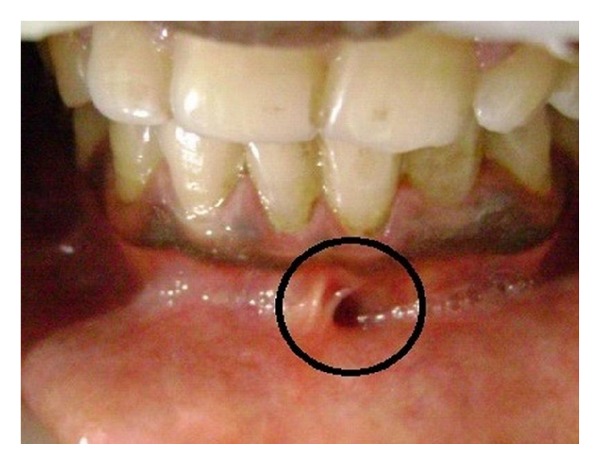
Clinical photograph showing draining sinus in relation to 31 and 32.

**Figure 2:**
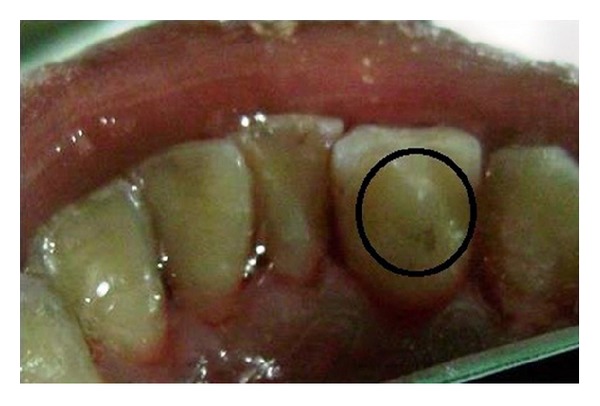
Clinical picture showing the talons cusp with foramen ceacum in relation to 32.

A young 14-year-old male patient reported to the Department of Pedodontics and Preventive Dentistry, with a chief complaint of pus discharge from the mandibular left anterior tooth region i.r.t. 31 and 32 for a week. He reported that he sustained an injury while brushing his teeth with a neem stick 4-5 months back. No relevant history of pain was reported. Thorough oral examination revealed a sinus in relation to tooth number 31 and 32 topographically. Tooth 32 had a clinical presentation of Talon's cusp with deep foramen caecum (not interfering with occlusion) [Figures 1 and 2]. The vitality test showed that the teeth was non vital.

**Figure 3:**
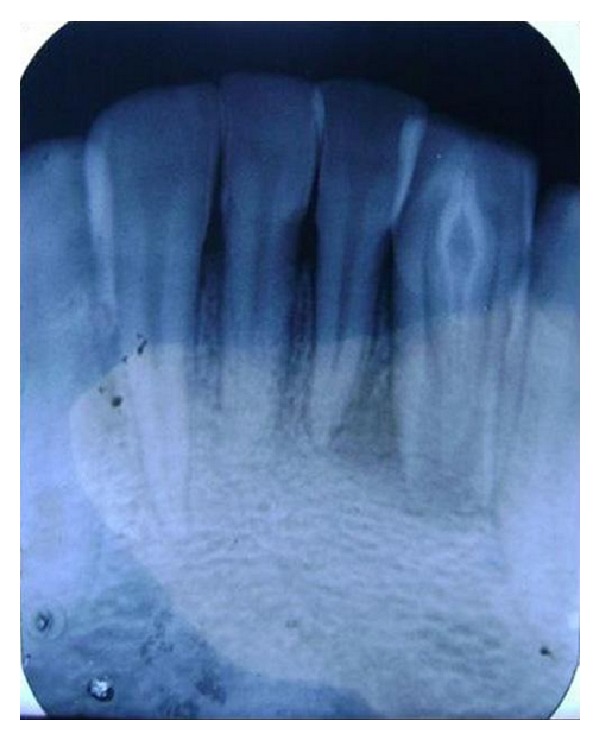
Initial radiograph showing DI type II with immature apex in relation to 32 and periradicular lesion associated with 31 and 32.

**Figure 4:**
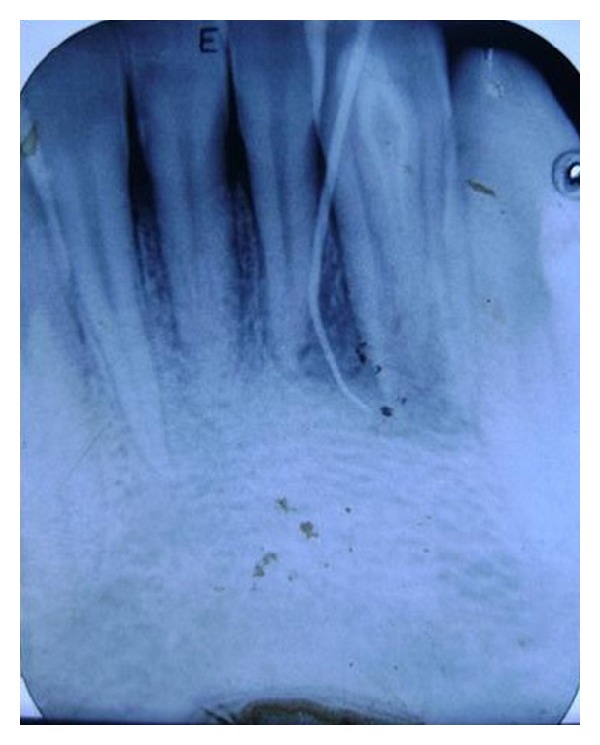
Radiograph showing GP cone pointing to the involvement of 31 and 32.

A diagnostic periapical radiograph revealed that 32 presented with dens in dente type II with immature apex and periapical radiolucency of approximately 15 mm × 20 mm extending distal to the root tip of tooth number 31 [Figure 3]. Another radiograph was taken with a gutta percha point placed through the sinus (through and through connection). Endodontic treatment of the involved teeth (31 and 32) was the plan of treatment [Figure 4].

**Figure 5:**
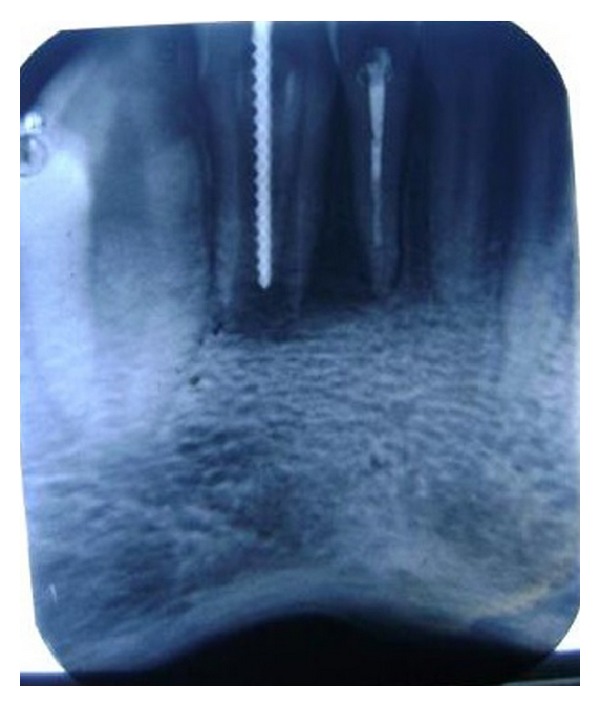
Radiograph showing the whole removal of parts of invagination and rudimentary canal in relation to 32 and metapex dressing in 31.

With emergency treatment the abscess was drained through the vestibular sulcus. Antibiotics and analgesics were prescribed. Six days later the patient was recalled again and Talons cusp in relation to 32 was grinded to expose the pulp. A large central canal was found but there was no communication between the mesial and distal rudimentary canals in the DI. This was confirmed by the radiographic dye. Then complete elimination of invagination was done with the aid of a K-Flex #80 file [Figure 5]. In the same appointment, access preparation in 31 was done. Treatment was performed under local anaesthesia under complete rubber dam isolation.

**Figure 6:**
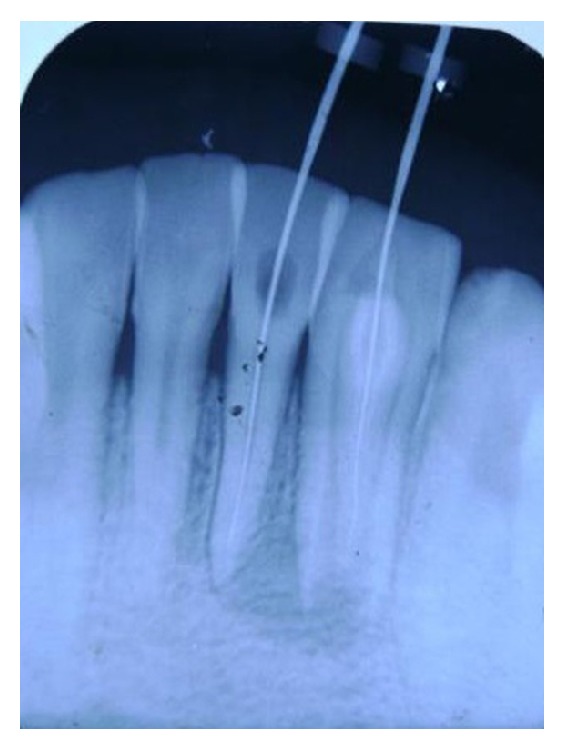
Radiograph reveals working length determination of 31 and 32.

After establishing optimal working length in the radiograph of 31 and 32, the canal was disinfected with 5.25% sodium hypochlorite [Figure 6]. Then canals were dried with absorbent points. Calcium hydroxide was placed as medication inside the canal of 32. This was changed every 2 weeks for a period of 2 months. Metapex was placed in 31. There were signs of healing of the periradicular tissue.

**Figure 7:**
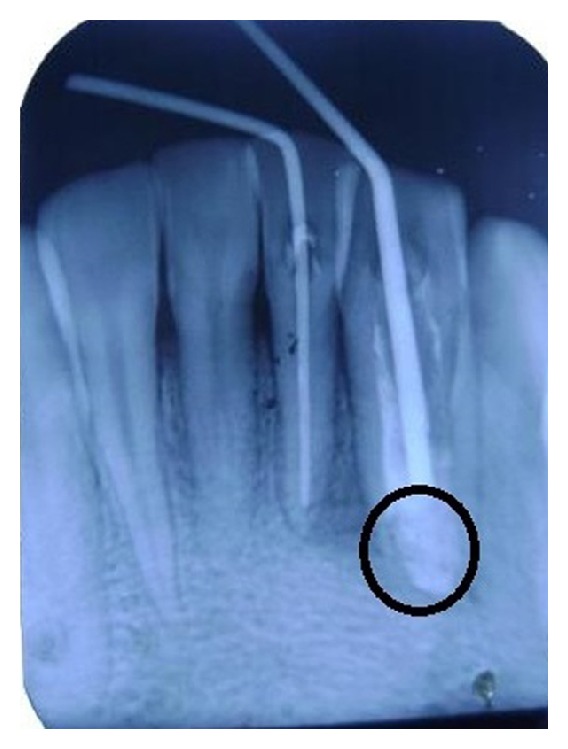
Radiograph showing apical plug of 4 mm MTA in relation to 32 and master cone in 31.

**Figure 8:**
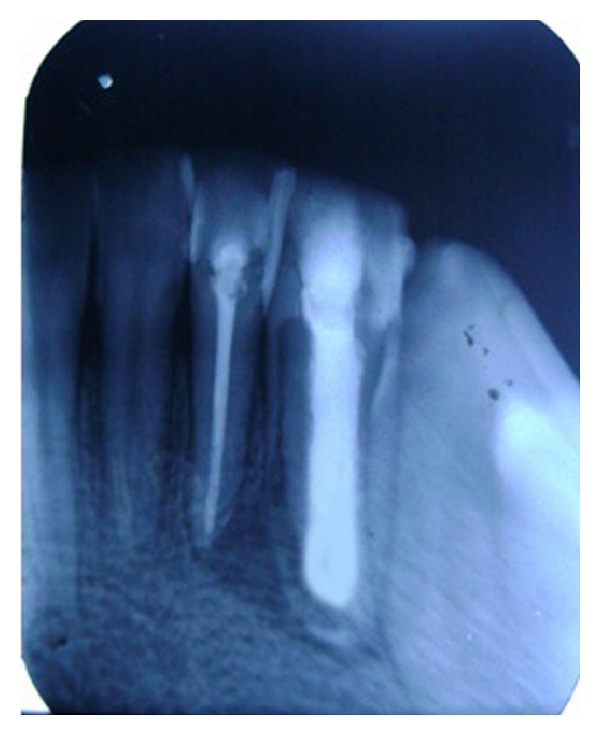
Root canal of 31 and 32 obturated with GP along with GIC lining in relation to 32 (dentinal wall) and completed healing of PA tissues.

An apical barrier of 4 mm in thickness was created with Pro Root MTA (Dentsply Tulsa Dental Company) in 32 and it was left with a cotton pellet moistened with distilled water for 48 hours [Figure 7]. At the following appointment, the cotton pellet was removed and after verifying the setting of MTA, the wall of the canal was first coated with a layer of glass ionomer cement (GIC) as the canal was large. GIC was used to maintain tooth resistance to fracture. After 2 months of dressing with metapex, 31 were obturated with the help of gutta-percha points [Figure 8].

The patient was recalled for regular clinical visits at six monthly intervals and there was a complete absence of clinical symptoms.

## 3. Discussion

An early diagnosis of dens in dente is crucial and requires a thorough clinical examination of all teeth. Radiographically, this anomaly demonstrates a radio opaque invagination, equal in density to enamel and extending from the cingulum into the root canal. In order to characterize the degree of extent in the crown, root, and up to apex, Oheler classified DI as follows: [9]

1. type I—an enamel invagination within the crown and not extending beyond the CEJ,
2. type II—the enamel invagination into the root below the CEJ ending as a blind sac,
3. type III—the extension of enamel-lined invagination through the root to form an additional apical or lateral foramen; usually no direct communication with the pulp.

The presumed etiology of this phenomena has been related to focal growth retardation, rapid and aggressive proliferation of a part of the inner enamel epithelium, growth pressure of the dental arch, localized external pressure in certain areas of the tooth bud, distortion of the enamel organ, fusion of the two tooth germs, infection, and trauma [10].

The clinical appearance of dens invaginatus varies considerably. The crown of the affected tooth can be of normal morphology but also can be associated with unusual forms like greater labiolingual diameter, peg shaped, barrel shaped, conical shaped, or talons cusp [6, 11]. As in our case the clinical appearance of talons cusp and periapical radiograph showed an invagination into pulp which extended into the root of the left mandibular lateral incisor which was categorized as Oehlers type II dens invaginatus.

Depending on the degree of malformation of DI and presence of clinical symptoms, there are different treatment modalities to prevent possible complications. Even without symptoms, dental treatment is considered necessary because access of irritants to the invaginatus may result in immediate or eventual contact with pulp. Strict observation is recommended if dental treatment is not done. The invaginatus is usually removed during treatment as was also done in this case.

Various techniques of treating DI have been reported including conservative restorative treatment, teeth with deep palatal or incisal invagination or foramen caecum treated with fissure sealants before carious destruction can occur, nonsurgical root canal treatment, endodontic surgery, and intentional replantation and extractions [12]. Nonsurgical root canal therapy has been considered impractical with type II and type III dens invaginatus because of unpredictable internal anatomy and the challenge in inadequately cleaning without removing the dens. In the past, removal of dens was difficult but with the advancement of technology and operating instruments this option has been made possible [13].

A complete disinfection of the canal is of great importance to promote healing of affected periradicular tissues. In this case, sodium hypochlorite as irrigation and calcium hydroxide and metapex as intracanal medication between appointments were used to obtain this result.

One of the major problems in endodontic therapy in teeth with pulp necrosis and open apex is obtaining an adequate closure of the root canal. The clinical procedure required for these teeth is based on the principle of pulp space disinfection allowing the formation of a mineralized tissue barrier at the apex of the tooth. Calcium hydroxide is the most commonly used medication for this purpose.

MTA has been proposed as a material for immediate closure of the apical opening without waiting for a natural healing process. This will create an apical barrier in the canal preventing the extrusion of root filling material into the periapical tissues [14]. It has been demonstrated that MTA induces the formation of a calcified matrix in the periapical tissue and the regeneration of new cement, possibly associated with its high sealing capacity, biocompatibility, alkaline pH, and liberation of substances activating the cementoblasts, which in turn will deposit a matrix for the cementogenesis [15, 16].

The dens invaginatus type II reported here had a diagnosis of chronic periapical abscess with immature apex. In our case, the root canal was filled apically with an approximate thickness of 4 mm of MTA. At a later appointment the rest of the root canal was coated with glass ionomer as a liner and obturated with gutta percha. This was an attempt to reinforce the root, as there is a high incidence of root fracture after endodontic treatment in these types of teeth with large pulp space [17].

Sathorn and Parashos reported a case of type II DI of maxillary canine in which the dense and infected necrotic pulp were removed and apexification with MTA was done [13]. Kristoffersen et al. reported an immature maxillary right lateral incisor with type II DI in which dens was removed, calcium hydroxide applied as intracanal medicament, and MTA was used as an apical barrier [18]. Similar technique, and findings were also reported in other studies [2, 10, 11, 19].

Our case is unique by itself because the patient was unaware of the presence of the tooth malformation related lesion. DI presented as a talons cusp on the mandibular lateral incisor which is a rare anomaly. Radiographic evidence presented that the tooth was involved in periradicular infection, and hence root canal therapy was advised but at the same time the preservation of resistance of tooth structure was also kept in mind.

## 4. Conclusion

This anomaly is clinically important because potential complications lead to pulp necrosis and periapical infection. A severe form often causes periapical cyst formation at an early age. As this is a rare phenomenon, it is necessary for clinical and radiographic assessments to identify the defects. Appropriate care has to be taken for the intervention so as to be sure of a favourable prognosis.

## References

[1] Hülsmann M (1997). Dens invaginatus: aetiology, classification, prevalence, diagnosis, and treatment considerations. *International Endodontic Journal*.

[2] Silberman A, Cohenca N, Simon JH (2006). Anatomical redesign for the treatment of dens invaginatus type III with open apexes: a literature review and case presentation. *The Journal of the American Dental Association*.

[3] Zengin AZ, Sumer AP, Celenk P (2009). Double dens invaginatus: report of three cases. *The European Journal of Dentistry*.

[4] Hovland EJ, Block RM (1977). Nonrecognition and subsequent endodontic treatment of dens invaginatus. *Journal of Endodontics*.

[5] Mupparapu M, Singer SR (2004). A rare presentation of dens invaginatus in a mandibular lateral incisor occurring concurrently with bilateral maxillary dens invaginatus: case report and review of literature. *The Australian Dental Journal*.

[6] Reddy YP, Karpagavinayagam K, Subbarao CV (2008). Management of dens invaginatus diagnosed by spiral computed tomography: a case report. *Journal of Endodontics*.

[7] Munir B, Tirmazi SM, Majeed HA, Khan AM, Iqbalbangash N (2011). Dens invaginatus: aetiology, classification, prevalence, diagnosis and treatment considerations. *The Pakistan Oral and Dental Journal*.

[8] Tulunoglu O, Canaka DU, Ozdemir RC (2007). Talon’s cusp: report of four unusual cases. *Journal of Indian Society Pedodontics and Preventive Dentistry*.

[9] Oehlers FAC (1957). Dens invaginatus (dilated composite odontome). I. Variations of the invagination process and associated anterior crown forms. *Oral Surgery, Oral Medicine, Oral Pathology*.

[10] Fregnani ER, Spinola LFB, Sônego JRO, Bueno CES, de Martin AS (2008). Complex endodontic treatment of an immature type III dens invaginatus: a case report. *International Endodontic Journal*.

[11] Jung M (2004). Endodontic treatment of dens invaginatus type III with three root canals and open apical foramen. *International Endodontic Journal*.

[12] Tewari S, Malhotra ML, Goel VP, Maheshwari PK (1992). A rare variety of coronal type of dens invaginatus. *The Journal of the Indian Dental Association*.

[13] Sathorn C, Parashos P (2007). Contemporary treatment of class II dens invaginatus. *International Endodontic Journal*.

[14] Torabinejad M, Watson TF, Pitt Ford TR (1993). Sealing ability of a mineral trioxide aggregate when used as a root end filling material. *Journal of Endodontics*.

[15] Torabinejad M, Hong C-U, Lee S-J, Monsef M, Pitt Ford TR (1995). Investigation of mineral trioxide aggregate for root-end filling in dogs. *Journal of Endodontics*.

[16] Torabinejad M, Pitt Ford TR, McKendry DJ, Abedi HR, Miller DA, Kariyawasam SP (1997). Histologic assessment of mineral trioxide aggregate as a root-end filling in monkeys. *Journal of Endodontics*.

[17] Frank AL (1966). Therapy for the divergent pulpless tooth by continued apical formation. *The Journal of the American Dental Association*.

[18] Kristoffersen Ø, Nag OH, Fristad I (2008). Dens invaginatus and treatment options based on a classification system: report of a type II invagination. *International Endodontic Journal*.

[19] Lichota D, Lipski M, Woźniak K, Buczkowska-Radlińska J (2008). Endodontic treatment of a maxillary canine with type 3 dens invaginatus and large periradicular lesion: a case report. *Journal of Endodontics*.

